# Developing an evidence-informed framework for safe and accessible urban mobility infrastructures for older adults in low- and middle-income countries: a protocol for realist synthesis

**DOI:** 10.1186/s13643-020-01456-w

**Published:** 2020-08-24

**Authors:** Divya Sussana Patil, Uday Narayan Yadav, Sobin George, Marco Helbich, Dick Ettema, Ajay Bailey

**Affiliations:** 1 Transdisciplinary Centre for Qualitative Methods, Department of Health Information, Prasanna School of Public Health, Manipal Academy of Higher Education, Manipal, India; 2 Centre for Primary Health Care and Equity, University of New South Wales, Sydney, Australia; 3 Centre for Study of Social Change and Development, Institute for Social and Economic Change, Bengaluru, Karnataka India; 4 Department of Human Geography and Spatial Planning, Faculty of Geosciences, Utrecht University, Utrecht, The Netherlands

**Keywords:** Older adults, Mobility infrastructures, Transportation, Transport interventions, Urban mobility

## Abstract

**Background:**

Mobility, one of the basic daily activities, helps in carrying out routine work, which contributes to people’s well-being. A lack of friendly and accessible infrastructure may act as a barrier, which limits older adults’ contributions and participation in society. Hence, it is important to have an enabling environment for older adults to carry out their activities independently at ease. There is ample research evidence about effective interventions on urban mobility infrastructures, but there is a lack of evidence regarding what works, for whom, and in what circumstances. Hence, there is a need to identify the contextual factors for different regions to design region-specific interventions. The aim of this realist synthesis is to develop an evidence-informed framework for safe and accessible urban mobility infrastructures for older adults in low- and middle-income countries.

**Methods:**

A realist review will be undertaken using the following process: (1) development of a program theory, (2) search strategy and information sources, (3) study selection and appraisal, (4) data extraction, and (5) data synthesis. In addition to searching grey literature and contacting authors, we will search (since inception) multiple electronic databases such as PubMed, EMBASE, Scopus, Web of Science, and Cochrane Library. Studies will be included based on their ability to provide data that evaluates some aspect of the program theory. Two independent reviewers will screen and extract data from all relevant sources. A realist logic of analysis will be used to identify all context-mechanism-outcome that explains how safe and accessible urban mobility infrastructures for older adults implemented in low- and middle-income countries translate to better health outcomes. The findings will be reported according to Realist and MEta-narrative Evidence Syntheses: Evolving Standards guidelines.

**Discussion:**

This realist review will help to develop a framework for safe and accessible urban mobility infrastructures for older adults in low- and middle-income countries. The results of this study will support evidence-based decision-making on urban mobility systems and will be of interest to various stakeholders. Dissemination will be done through conference presentations, policy briefs, media, and peer-reviewed journals. Implications for future research will be discussed.

**Systematic review registration:**

PROSPERO CRD42020168020

## Background

Residential neighborhoods as well as important destinations outside the neighborhood that are age-friendly are places that promote healthy aging and well-being of older adults (OAs). The ability to move around or use public transport is among the major factors supporting healthy aging. Mobility helps to carry out daily routines around the house, to access services in the community, to visit healthcare facilities, and to participate in socio-cultural activities. Owing to the rise in global population aging, there is a growing need for various facilities in the society such as goods and services, transportation, housing, and social protection [1].

In accordance with the United Nations (2017), the global figure of OAs was approximately 962 million, which comprises 13% of the world’s population. The majority of growth in the population of OAs will occur in the Global South in the coming years [1]. The World Health Organization (WHO) estimates that by 2050, 80% of the OAs will be living in low- and middle-income countries (LMICs) [2]. In 1950, the population of OAs in southern Asia was 5.8%, which increased to 8.4% in 2015 and will reach 12% by 2050 [3]. About 8% of the Indian population are OAs, which is projected to be 19% by 2050 [4]. Since the population of OAs is growing at an increasingly fast pace, it would be necessary to ensure progress towards the goals outlined in Sustainable Development Goals (SDG). The SDG 3 aimed to ensure healthy lives and promote well-being for people of all ages, and SDG 11 emphasizes on making cities sustainable by creating career opportunities, providing safe and affordable housing, investing in public transport, creating green public spaces, and improving the urban planning and management [5]. To achieve these goals, while LMICs are speeding up the infrastructure development project, there is a lack of clear and context-specific evidence-based decision-making framework on urban mobility systems focusing on older adults.

Reduced mobility within the community can have an impact on the quality of life of older adults. They face many barriers in the community that relate to the built environment, which includes public transportation and infrastructure [6]. Visiting distant places could become a challenge to those not having private vehicles due to a lack of access to efficient public transportation and inadequate pedestrian facilities. Even if there is transportation, timing and frequency may not be convenient for older adults. A review conducted in rural Michigan showed that there was a lack of suitable pedestrian pathways, roads, and disabled-friendly lanes/access ramps, suggesting an inequitable distribution of facilities for vulnerable population like OAs [7, 8]. Analysis from a study conducted in the UK and Austria showed that the quality of physical infrastructure, issues around implementation of transport interventions, and services and attitude or behavior of staff were important factors affecting mobility among OAs [9, 10]. Ways to improve access to transportation include increasing the convenience by changing the routes/timetables, offer subsidized fares or free passes for OAs, and priority seating arrangements, and to educate the transport staff to be more considerate towards OAs who may need some help or take more time to get on and off the public transport [11].

Another review conducted globally identified that longer distance from the origin point, i.e., home to access public transport; difficulty to enter and exit the vehicle; lack of respect from drivers; irregular timing of public transport; insecurity while traveling alone; transportation fare; no information displays; lack of proper seating arrangements; improper bus shelters; and dim-lit subways and pathways were barriers to public transport for the older adults [8]. These barriers need to be addressed to make public transportation and infrastructure in which people live and work accommodating to the OAs. However, the review did not identify contextual factors specific to countries based on income categories. A report on age-friendly cities by the World Health Organization shows that accessibility, reliability, frequency, safety, availability, and affordability is not a challenge in most of the developed countries, whereas it is a concern expressed by OAs in developing countries [12]. This report indicates that attention should be given to developing a sustainable transport system, which will be accommodating for OAs in developing countries.

There is evidence from other developed countries in Europe that have become age-friendly and ideas from such transport interventions can be considered in developing nations as well [13]. However, it is important to understand that there are economic and political barriers that could limit the implementation of interventions in certain developing countries [14]. Lack of funding and expertise in the field of urban transportation can be one of the reasons for inadequate urban mobility infrastructure [15]. A systematic review of interventions like urban regeneration, improving green infrastructure, and improving transport infrastructure showed that there is an improvement in mental health and quality of life outcomes to some extent [16]. A study conducted in Bangkok showed that accessible transportation services to public spaces, ability to travel independently, and patterns of urban development factors affected the mobility of OAs [17]. Another case study from Delhi, India, suggested that having properly designed road infrastructure for non-motorized and public transport will benefit in reducing road congestion, transporting more people at the same time, reduces commute time, and increases safety. While there is ample evidence about effective interventions, there is a lack of evidence regarding what works, for whom, and in what circumstances with respect to OAs in LMICs. Transport infrastructure interventions are complex, and their impact on the well-being of OAs depends on contextual factors such as geographical, socio-cultural, political, and socio-economic factors. Hence, it is necessary that contextual factors should be identified for different regions in order to design region-specific interventions. More often, evidence obtained from traditional methods of review does not give us insights into the effect of specific local contextual factors and on the effectiveness of interventions focusing on OAs. Until now, there has been no systematic evaluation of the underlying mechanisms in interventions for safe and sustainable urban mobility systems in LMICs. Traditional systematic reviews generally consider randomized controlled trials and quasi-experimental research designs to have a meta-analysis of the effectiveness of interventions as the output. Such approaches are likely masking that interventions may or may not work for certain population subgroups under certain circumstances. However, realist synthesis is particularly suitable to document best practices as it includes a broad range of studies having the aim to identify how, why, for whom, and in what context social interventions work. The purpose of this realist synthesis is to develop an evidence-informed framework for interventions in low- and middle-income countries (LMICs) to improve safety and access in urban mobility infrastructure by understanding what works, for whom, and in what context. This research involves a realist approach, which can help us develop novel insights into urban mobility infrastructure. The research questions are as follows: (1) What are the underlying mechanisms of mobility infrastructure interventions in low- and middle-income countries to improve the accessibility and safety of older adults? and (2) How context such as geographical location, socio-cultural, socio-economic, and political factors influences the success or failure of such interventions?

## Methods

The review protocol has been registered within the PROSPERO database with registration number CRD42020168020 and is being reported in accordance with the reporting guidelines provided in the Preferred Reporting Items for Systematic Reviews and Meta-Analyses Protocols (PRISMA-P) statement [18, 19] (see checklist in Additional file 1). The proposed realist review will be reported in accordance with the reporting guidance provided in the Realist and MEta-narrative Evidence Syntheses: Evolving Standards [RAMESES II] [20].

A realist synthesis [21] is a relatively new approach to synthesize evidence, which has an explanatory approach rather than a judgmental approach. Unlike the traditional systematic reviews, it seeks to explain the mechanisms for how complex interventions work or fail in particular real-world settings. Pawson and colleagues stress that, in order to understand what works in certain interventions involves establishing a causal relationship between activities and the intended outcome. Moreover, middle-range theories are an outcome of a realist synthesis, where the context-mechanism-outcome (CMO) configuration in a program will help us to understand how, why, and for whom an intervention produced the desired and undesired outcomes. The middle range theory in the context of this research will be the “evidence-informed framework.” It is used in complex social interventions and is based on a realist approach rooted in sociology [21]. The steps involved in a realist synthesis include (1) developing the program theory; (2) search strategy and information sources; (3) screening, study selection, and appraisal; (4) data extraction; (5) data synthesis; and (6) stakeholder consultation and refinement of program theory.

### Developing the program theory

To develop a program theory, a broad search for literature will be undertaken to identify interventions to improve safety and access to urban transport for OAs, where they were implemented and what were the expected outcomes. The preliminary search for literature showed that there were different interventions undertaken with respect to urban transport infrastructure, its safety, and transport policies. After identifying the intervention strategies, the program theory will be developed, which will help us understand what mechanisms influence in having safe and accessible urban transport for OAs. For example, subsidies in fares help increase the usage of public transport. Similarly, interventions to make the transport infrastructure more accessible and safer will increase the usage of public transport by OAs. Hence, we can assume that having an efficient transport infrastructure may help OAs have better access to healthcare, essential services, and social life thus improving their well-being. We will consult with multidisciplinary experts to get their inputs during the process to refine the program theory.

### Search strategy and information sources

Empirical evidence in order to refine the program theory will be searched. The search strategy and terms will be guided by the program theory. Search for literature will be conducted to identify the interventions on mobility infrastructure. Examples of population-specific keywords include “older adults,” “elderly,” and “older persons”; intervention-specific keywords include “mobility infrastructure,” “mobility interventions,” “urban transportation,” “transportation,” “mobility,” “transport infrastructure,” “transport services,” “public transport,” “pedestrian facilities,” and “walking pathway.” The search for literature will be designed and conducted by the review team. In order to have a comprehensive search strategy, we will consult an information scientist from the lead author’s institution to improve the search terms. The primary source of literature will be a structured search multiple electronic databases (from inception onwards): PubMed/MEDLINE, EMBASE, Scopus, Web of Science, Cochrane Library, ScienceDirect, ProQuest, REHABDATA, Transport Research International Documentation (TRID), and Mobility in Cities Database. The secondary source of potentially relevant material will be a search of the grey or difficult to locate literature, including Google Scholar and GreySource Index (GreyNet International, Amsterdam, The Netherlands). We will perform hand searching of the reference lists of included studies, conference proceedings, relevant review reports, dissertations, and theses. Content experts and authors who are familiar with the subject will be contacted. A draft search strategy for PubMed/MEDLINE and search keywords has been provided in part A of Additional file 2.

### Screening, study selection, and appraisal

Articles will be included in the review based on their relevance to provide information for the program theory. The inclusion of studies will be based on the following criteria: (1) articles related to urban transport infrastructure interventions aimed at improving older adults’ safety and accessibility; (2) should be conducted in low- and middle-income countries; (3) include all English and those non-English literatures, where the project team has the expertise to translate; (4) all types of articles without any restriction on publication type; and (5) outcomes should focus on the impact of urban transport infrastructure interventions on health and well-being. We will exclude studies that do not provide sufficient information on why an intervention worked, for whom, and in what context. Data will be managed using the Endnote software.

Screening will be undertaken based on the title, abstract, and full text by two reviewers. Those studies fulfilling all the criteria will be marked as “included,” and those not fulfilling all the criteria will be marked “excluded.” Studies for which there is no clarity will be marked as “unclear,” and any disagreements will be resolved by discussion with another expert until we reach consensus. The reasons for exclusion will be recorded. Quality appraisal for a realist synthesis will be done based on the relevance and rigor of the study. Relevance refers to how much a study contributed to building the theory, and rigor refers to how much the method used to generate data can be trusted. In addition, we will use quality assessment tools appropriately based on individual study design. For example, we will use “RoB 2.0 tool” for randomized trials [22], “Newcastle-Ottawa scale” for cross-sectional and cohort studies [23], “critical appraisal skills program” tool for qualitative studies [24], and “mixed methods appraisal tool” for mixed methods studies [25].

### Data extraction

Data will be extracted on a pre-designed, pilot-tested data extraction sheet by the lead author and will be checked by the second author. Any disagreements will be resolved by discussion with another reviewer until we reach consensus. The data extraction form will include the contents of the preliminary program theory. During data extraction, if any relevant information is missing in the article, it will be marked as “not reported.” The data extraction format will be pilot tested on two selected articles. The data extraction sheet will include study characteristics (title, author, publication year, publication status, country, participants, study objective), intervention-related details (relevance to program theory and implementation strategies), program theory (context, mechanism, and outcome aspects), and quality appraisal of the study. Draft data extraction sheet is available in part B of Additional file 2.

### Data synthesis

The analysis process in a realist review involves refining the already existing program theory to formulate an evidence-informed framework for effective intervention to improve the mobility and safety of OAs in urban mobility infrastructure. The analysis process will be done by a discussion with the review team. Once the data is extracted using the data extraction tool, it will be summarized into evidence tables. We will use both deductive and inductive analysis processes. Deductive codes will be developed based on the initial program theory. The extracted data will be coded, and additional themes relevant to the program theory will be developed. The results will be synthesized based on “what works,” under “what circumstances,” and for “whom.” The results will be presented narratively.

### Stakeholder consultation and refinement of the program theory

Including stakeholders in research is important in policymaking and is recommended for a realist synthesis [26, 27]. It will help to develop and refine program theories. Hence, interviews/discussion will be conducted with different stakeholders such as transport policymakers, urban transport planners, older adults, transport intervention implementers, public transport specialists, public relation officers, user groups, and engaged public in order to obtain a wide range of perspectives regarding improving access to urban mobility infrastructures.

We will develop an evidence-informed framework for effective interventions to improve the mobility and safety of OAs in mobility infrastructure. Transport policymakers and program implementers can use this framework to design region-specific interventions. The research focuses on co-designing an inclusive urban mobility evaluative framework that can provide guidelines for making urban transport infrastructures and services more inclusive towards marginalized and vulnerable groups.

## Discussion

Many governments in LMICs are making efforts to improve the mobility infrastructure for older adults, particularly in urban areas. In this line, our well-informed recommendations grounding on evidence will guide the relevant stakeholders to develop the interventions, which would be simpler as to *why* an intervention worked better in specific settings, and how context can influence the effectiveness of interventions. Moreover, our review will help to build a realist program theory and will identify the priorities, which may guide future evidence-based primary research in LMICs and would inform future policy, research, and practice aimed to develop sustainable urban mobility interventions for the older population.

The anticipated challenge, which is usually seen in realist reviews, is the inadequacy of available literature to come up with program theory. In order to address this issue, we have team members with expertise in search (DSP and UNY), and we will use the information from stakeholder interviews to get additional data relevant to the review question. Another important challenge is regarding the rigor of the selected studies. We will address this limitation by considering how the study was conducted and reported and by identifying any limitations in the final analysis. We will also consider the comments received from different stakeholders during the review process. It is possible that relevant studies and gray literature will be in non-English languages. We will seek the help of bilingual experts or translators as far as possible to overcome this challenge. In several countries, local urban bodies are responsible for the urban transport interventions, and their reports are not always accessible in the public domain. However, we will attempt to access such gray reports wherever possible. It is also possible that interventions of urban bodies may not be properly recorded or reported, which can be a limitation of the present exercise. Findings will be disseminated through publications in peer-reviewed journals, conference presentations, and knowledge exchange with stakeholders such as urban planners, transport department, transport policymakers, and older adults. Any amendments made to this protocol when the study is being conducted will be outlined and reported in the final manuscript.

## Supplementary information

**Additional file 1.** PRISMA-P Checklist.

**Additional file 2.** Search strategy for PubMed/MEDLINE and data extraction sheet.

## Data Availability

Data sharing is not applicable to this article as no datasets were generated or analyzed during this current study.

## Abbreviations

CMO: Context-mechanism-outcome
LMICs: Low- and middle-income countries
OAs: Older adults
PRISMA-P: Preferred Reporting Items for Systematic Reviews and Meta-Analyses Protocols
RAMESES: Realist and MEta-narrative Evidence Syntheses: Evolving Standards
RoB: Risk of bias
SDG: Sustainable Development Goals
TRID: Transport Research International Documentation
WHO: World Health Organization
