# Supplementary material for: Developing an evidence-informed framework for safe and accessible urban mobility infrastructures for older adults in low- and middle-income countries: a protocol for realist synthesis
Source: Syst Rev. 2020 Aug 24;9:196. doi: 10.1186/s13643-020-01456-w (PMC7445900; doi:10.1186/s13643-020-01456-w)
Supplement: Supplementary file 2 — Additional file 2. Search strategy for PubMed/MEDLINE and data extraction sheet. [file 13643_2020_1456_MOESM2_ESM.docx]

**Part A: Search keywords:** The keywords will be modified for different databases as required

| **Population**  “old* people” OR “old* persons” OR “older adults” OR “elder*” OR “elder* persons” OR “aged people” OR “aged population” OR “aging population” OR “ageing” OR “aging” |
| --- |
| **Intervention**  “Transport* infrastructure” OR “mobility infrastructure” OR “transport* intervention” OR “public transport” OR “public transit” OR “urban mobility infrastructure” OR “Motorized transport” OR “Non-motorized transport” OR “transport* planning” OR “transport* design” OR “modes of transport” OR “means of transport” OR “a form of transportation” OR “electric motor” OR “road transport” OR “roadways vehicle” OR “municipal transport system” OR “metropolitan transport system” OR “municipal mobility” |
| **Outcome**  “Well-being” OR “improved transport*” OR “quality of life” OR “health” |

**Draft search strategy for PubMed:**

| Search | Details | Results |
| --- | --- | --- |
| #1 | "Transport* infrastructure" OR "mobility infrastructure" OR "transport* intervention" OR "public transport" OR "public transit" OR "urban mobility infrastructure" OR "Motorized transport" OR "Non-motorized transport" OR "transport* planning" OR "transport* design" OR "modes of transport" OR "means of transport" OR "road transport" OR "roadways vehicle" | 192,185 |
| #2 | "Well-being" OR "improved transport*" OR "quality of life" OR "health" | 5,033,997 |
| #3 | "old* people" OR "old* persons" OR "older adults" OR "elder*" OR "elder* persons" OR "aged people" OR "aged population" OR "aging population" OR "ageing" OR "aging" | 1,389065 |
| #4 | Search: #1 AND #2 AND #3 Filters: Humans, Dutch, English, Middle Aged: 45-64 years, Aged: 65+ years, 80 and over: 80+ years | 1403 |

For Google scholar search, each concept in set #1 will be searched separately by combining them with concepts of #2 or #3. The first 30 articles from each search will be downloaded.

**Part B: Draft data extraction sheet for realist review (This will be piloted and refined as the review progresses)**

| Date | | Reviewer’s Initials | |
| --- | --- | --- | --- |
| Title: | | | |
| Author and year: | | | |
| Country: | | | |
| Participants: | | | |
| Objectives: | | | |
| Intervention: | | | |
| Implementation strategy(s): | | | |
| For what aspect(s) of program theory does this study provide evidence (i.e. evidence ‘to support, refute or refine elements of theory)? | | | |
| CMO Table (Or elements of CMOs) (Add extra lines if required). Include quotes or page no’s | | | |
| Context | Mechanism | | Outcome |
|  |  | |  |
|  |  | |  |
|  |  | |  |
| Methodology  Rigour:  Relevance: | | | |
| Other notes/comments | | | |
| Decision | | | |
